# Estimation of Inhibitory Effect against Tyrosinase Activity through Homology Modeling and Molecular Docking

**DOI:** 10.1155/2015/262364

**Published:** 2015-12-15

**Authors:** Daungkamon Nokinsee, Lalida Shank, Vannajan Sanghiran Lee, Piyarat Nimmanpipug

**Affiliations:** ^1^Computational Simulation and Modelling Laboratory (CSML), Department of Chemistry and Center of Excellence for Innovation in Chemistry, Faculty of Science, Chiang Mai University, Chiang Mai 50200, Thailand; ^2^Drug Design and Development Research Group, Department of Chemistry, Faculty of Science, University of Malaya, 50603 Kuala Lumpur, Malaysia

## Abstract

Tyrosinase is a key enzyme in melanogenesis. Generally, mushroom tyrosinase from* A. bisporus* had been used as a model in skin-whitening agent tests employed in the cosmetic industry. The recently obtained crystal structure of bacterial tyrosinase from* B. megaterium* has high similarity (33.5%) to the human enzyme and thus it was used as a template for constructing of the human model. Binding of tyrosinase to a series of its inhibitors was simulated by automated docking calculations. Docking and MD simulation results suggested that N81, N260, H263, and M280 are involved in the binding of inhibitors to mushroom tyrosinase. E195 and H208 are important residues in bacterial tyrosinase, while E230, S245, N249, H252, V262, and S265 bind to inhibitors and are important in forming pi interaction in human tyrosinase.

## 1. Introduction

Tyrosinase is a metalloprotein belonging to type 3 copper enzyme family. It is involved in melanin production in a wide range of organisms. The enzyme has a bifunctional catalytic mechanism consisting of the hydroxylation of monophenols to* o*-diphenols (monophenolase or cresolase activity) and the oxidation of* o*-diphenols to* o*-quinones (diphenolase or catecholase activity). Polymerization of products leads to melanin formation [1–3]. Tyrosinase is classified into three different oxidation states with different functions. Each copper atom for all forms is coordinated by three histidine residues. The first, oxy-form (oxy-tyrosinase or E_oxy_: [Cu(II)–O_2_^2−^–Cu(II)]) tyrosinase contains two tetragonal copper(II) ions, and dioxygen is bound as a peroxide molecule and acts as a bridge between two copper ions, in the oxidation state, and can react with monophenol or diphenol substrate. The oxy-tyrosinase can be obtained from met-tyrosinase by addition of hydrogen peroxide and deoxy-tyrosinase can be generated by binding to dioxygen. The met-form (met-tyrosinase or E_met_: [Cu(II)–Cu(II)]) tyrosinase contains two tetragonal copper(II) ions similar to oxy-tyrosinase and can react with diphenol to produce* o*-quinone. The met-tyrosinase can be converted to deoxy-tyrosinase by reducing copper(II) ions to copper(I) ions. Last, deoxy-form (deoxy-tyrosinase or E_deoxy_: [Cu(I)–Cu(I)]) tyrosinase can bind oxygen molecule and be reduced to oxy-form [4–6]. The active site of tyrosinase is characterized by two copper atoms (CuA and CuB) that are surrounded by a bundle of 4 helices and coordinated by six histidine residues. Copper is essential for the catalytic activity of tyrosinase. The active site is well conserved in diverse species [7].

Tyrosinase is a key enzyme in melanogenesis, which is essential for pigmentation. The catalysis of L-tyrosine to L-dopa is the rate-limiting step of the enzymatic pathway in melanin formation. Tyrosinase is also an important factor in wound healing and cuticle formation in arthropods and browning in plants [8–10]. In humans, melanin helps protect skin from the damage caused by ultraviolet radiation [11]. However, the excess level of melanin can cause various dermatological disorders including hyperpigmentations and is also linked to Parkinson's and other neurodegenerative diseases [12–15]. The disorders due to high levels of tyrosinase can be treated by tyrosinase inhibitors. Apart from importance of tyrosinase inhibitors in medicine, tyrosinase inhibitors attract much attention in the cosmetic industry as global market demand has increased for skin-whitening agents for individuals who want to obtain lighter skin color. Most tyrosinase inhibitors have been tested with commercial mushroom tyrosinase for use against mammalian tyrosinases. However, recent research has reported significant differences in inhibitor effectiveness between mushroom tyrosinase and human tyrosinase [16–19]. Thus, it is important to investigate an alternative model like those from bacteria for more accurate inhibitor screening. The amino acid sequences of tyrosinase of* Bacillus megaterium* were first reported in 2009 [20]. The tyrosinase gene of* Bacillus megaterium* was overexpressed in* E. coli* and purified using affinity chromatography. When its amino acid sequence was aligned with that of human tyrosinase, the result showed higher homology than that of* Agaricus bisporus*. For this reason, we aim to study structure of tyrosinase from three different sources including mushroom, bacteria, and human.

The crystal structure of* Agaricus bisporus* and that of* Bacillus megaterium* were reported in 2011 [4, 21]. The three-dimensional models constructed from the X-ray data are useful in catalytic mechanism studies of melanogenesis. From recent studies, the experimental results indicated different mechanisms between mushroom tyrosinase and human tyrosinase in regard to temperature, pH, *K*_*m*_ value, and IC_50_ value. The optimum temperature for L-dopa oxidation of human tyrosinase and mushroom tyrosinase was 50°C and 40°C, respectively. The optimum pH of human tyrosinase was more basic than mushroom tyrosinase. The *K*_*m*_ value for L-dopa was reported to be 0.31 mM for human tyrosinase and 1.88 mM for mushroom tyrosinase. The inhibitory effect of several tyrosinase inhibitors suggested that ascorbic acid was the best inhibitor of human tyrosinase and of mushroom tyrosinase as well when determined by the lowest IC_50_ values [17].

In this study, three-dimensional models of tyrosinase were studied focusing on binding structure with four common inhibitors: arbutin, ascorbic acid, kojic acid, and tropolone for screening and prediction of potent inhibitors of tyrosinases. The chelation of copper at the active site of the enzyme explains well the inhibitory effect of kojic acid, Chen et al., 1991 [22]. Arbutin is a glycosylated benzoquinone considered to be a nonphenolic agent. It is converted in the body to hydroquinone, a phenolic agent which inhibits the production of melanin [23]. Previous studies indicated that kojic acid, tropolone, and arbutin take part in chelation in inhibiting tyrosinase, while ascorbic acid reduces melanin formation via reduction of dopaquinone. In this way, precursor of the reaction will be perturbed. The homology model of human tyrosinase was generated using bacterial tyrosinase template which is more similar to human than the mushroom one. The key importance of our finding is to steer tyrosinase inhibitor discovery for therapeutic and cosmetic purposes. Structural information with the different models of tyrosinase, bacteria, mushroom, and human was investigated in this study. Our main interest was to propose that because bacterial tyrosinase has more similar structure to human tyrosinase, it should be used in both experimental and in silico screening of candidate molecules with potential tyrosinase inhibitory activity. The docking of tyrosinase was performed and key amino acids in binding pocket are highlighted.

## 2. Methods

### 2.1. Human Tyrosinase: Alignment and Homology Modeling

Homology modeling is the most reliable method for prediction of three-dimensional structures of unknown protein based on the assumption that the structure of the unknown protein is similar to the known structures of some homologous reference proteins [24]. When the three-dimensional structure of the human tyrosinase is identified, its model will be generated using the homology approach to evaluate its function. The complete amino acid sequence was retrieved from the National Center for Biotechnology Information (NCBI, http://www.ncbi.nlm.nih.gov/) [25] protein sequence database. A homolog protein template of the query protein human tyrosinase was identified by BLAST (Basic Local Alignment Search Tool) [26]. The protein template, tyrosinase of* B. megaterium* (3NQ1) with resolution at 2.3 Å, was selected because it has the highest known identity (33.5%) to the human enzyme. The structure of bacterial tyrosinase was determined [4]. The selected template and protein sequence were aligned to build a 3D structure using the Discovery Studio 2.5 software package [27, 28]. The structure was checked with PROCHECK [29] and Verify 3D [30, 31]. Energy criteria in comparison with the potential of mean force derived from a large set of known protein structures were determined.

### 2.2. Docking and Molecular Dynamic (MD) Simulations

To analyze binding scaffold of substrates and inhibitors with tyrosinase, molecular docking and dynamics simulations were carried out. Molecular docking was carried out using AutoDock4.0 software for prediction of binding structures of tyrosinase with inhibitors (ascorbic acid, arbutin, kojic acid, and tropolone). This software employs a semiempirical force field based on a comprehensive thermodynamic model and a Lamarckian genetic algorithm (LGA) for the conformational search [32]. The size of grid used in this work was set to be 60 Å × 60 Å × 60 Å in the *x*-, *y*-, and *z*-axis, respectively. A population size of 150 conformations and maximum number of energy evaluations of 2.5 million were applied. The cluster cut-off in groups was determined by RMSD value of 2 Å for comparison with the initial position of the starting ligand that was used. The conformation with the lowest energy in the highest numbered population size of cluster was selected.

Molecular dynamics (MD) simulations were performed using the AMBER 12 program [33]. The ff12SB force field was used for the structure of tyrosinase. Antechamber with the GAFF force field and Gasteiger charge were employed for ligands. All protein-ligand complexes were solvated in cubic box of TIP3P water extending at least 10 Å in each direction from the solute, while the cut-off distance was kept at 12 Å. All simulations were performed under periodic boundary conditions [34], and the long-range electrostatics force was treated by using the particle-mesh-Ewald method [35, 36]. Bond lengths involving those to hydrogen atoms were constrained using SHAKE. Prior to MD simulations, the systems were relaxed by a series of the steepest descent (SD) and conjugated gradient (CG). Minimizations of the MD simulations were performed based on each of the minimized systems by gradually heating over 60 ps from 0 to 300 K. In the following step, 1 ns MD equilibration was carried out employing time step of 2 fs. Finally, 10 ns MD simulations were conducted for each fully flexible system in the NPT at a constant temperature of 300 K. A total of 100 snapshots were extracted from the corresponding 300 ps of MD trajectories for binding free energy analysis.

## 3. Results and Discussions

### 3.1. Human Tyrosinase Model

**Figure 1:**
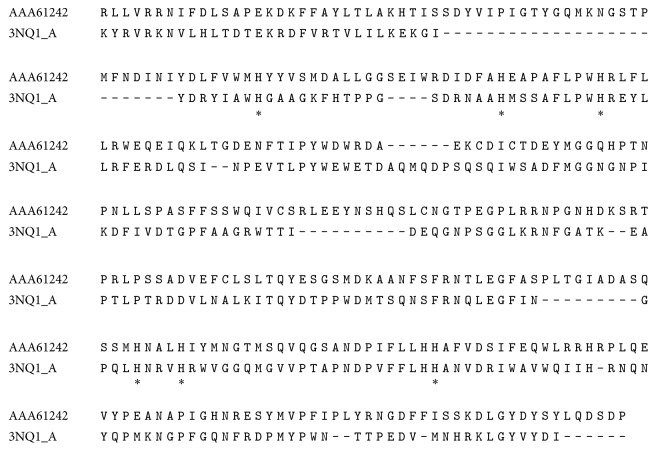
Sequence alignment between human amino acid sequence (AAA61242) and crystal structure of bacterial tyrosinase (3NQ1) with identity of 33.5% and similarity of 50.7%. Six histidine residues, which are provided by a four-helical bundle, coordinate the two copper ions (CuA and CuB) in the active site [1].

The crystal structure of* B. megaterium* tyrosinase with the highest resolution of 2.3 Å was selected. The sequence alignment between the query protein sequence (protein id = AAA61242) and template protein sequence (PDB id = 3NQ1) showed 33.5% identity (Figure 1). Six histidine residues in the active site of the target were matched with those of template. Matching residues were highlighted by star symbol.

**Figure 2:**
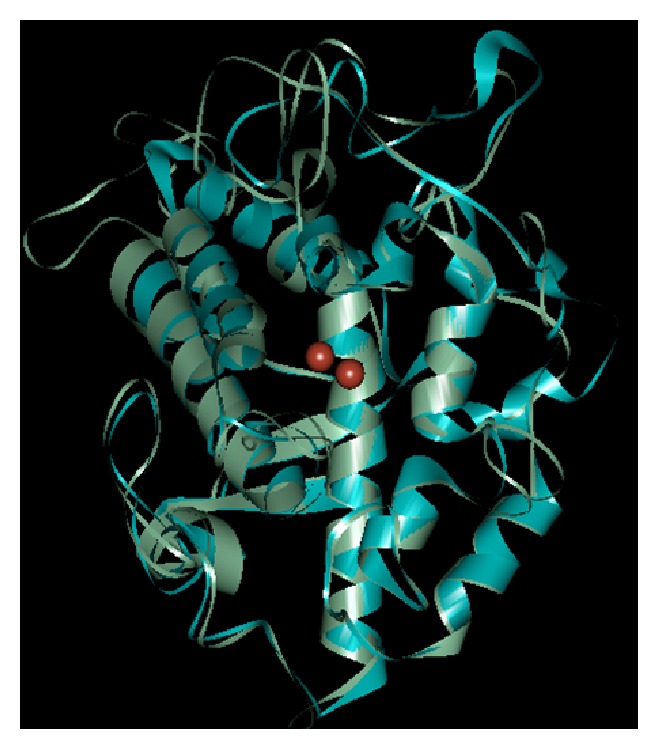
Superimposition of homology model (red) and its template (blue).

A superposition of the three-dimensional structures of the human homology model and that for* B. megaterium* tyrosinases shows slight deviations from its template of 0.64 Å (Figure 2).

**Figure 3:**
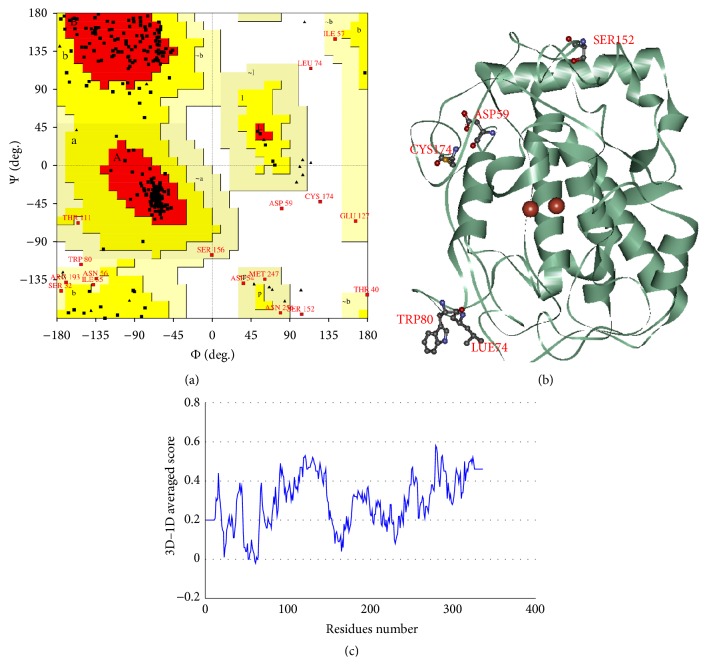
Quality of validation of the homology model. (a) Ramachandran plot of human tyrosinase. (b) Residues in disallowed region are Asp59, Lue74, Trp80, Ser152, and Cys174. (c) Verify 3D plot.

The model was validated using PROCHECK and Verify 3D. The Ramachandran plot of human tyrosinase (Figure 3(a)) indicated the most favored regions in red, and additional allowed, generously allowed, and disallowed regions are shown in yellow, light yellow, and white, respectively. There are 81.6% of residues in the most favored regions; 12.6% of residues are in additional allowed regions; 4.1% of residues are in generously allowed regions; and the remaining 1.7% are in disallowed regions. Residues in disallowed region are Asp59, Leu74, Trp80, Ser152, and Cys174 which are apart from the active area as shown in Figure 3(b). The Verify 3D plot (Figure 3(c)) shows a compatibility score of the model with its sequence. If more than 70% of the residues have a score of greater than or equal to 0.2, then the protein structure is considered to be of high quality. As shown in Figure 3(c), 70% of residues of the generated model have score over 0.2; thus, the quality of the predicted model is suitable for further analysis [31].

### 3.2. Binding Scaffolds: Docking and MD Simulation

Comparison of docking result with experimental data is shown in Table 1. Both L-tyrosine and L-dopa can bind with mushroom tyrosinase with essentially the same *K*_*m*_ value. Binding of substrate to bacterial and human tyrosinase indicated that L-tyrosine binds with both human and bacterial tyrosinases with higher affinity than does L-dopa. Binding energy analyzed from molecular docking was validated as shown in Table 1. The binding of L-tyrosine with mushroom and bacterial tyrosinase was compared. The simulated binding model was validated with experimental results at the same condition. The *K*_*m*_ value (at 25°C [20, 45]) and binding energy of L-tyrosine in complex with mushroom and bacterial tyrosinase are 0.2 mM and −10.00 kcal/mol and 0.075 mM and −11.09 kcal/mol, respectively. In case of L-dopa, mushroom tyrosinase has *K*_*m*_ value and binding energy of 0.17 mM and −10.20 kcal/mol, and of 0.35 mM and −10.05 kcal/mol for bacterial tyrosinase. The *K*_*m*_ value at 37°C [14] and binding energy of L-tyrosine and L-dopa with mushroom tyrosinase are 0.347 mM and −10.00 kcal/mol and 1.44 mM and −10.20 kcal/mol. In human tyrosinase, L-tyrosine and L-dopa have *K*_*m*_ value [2] and binding energy of 0.17 mM and −11.66 kcal/mol and 0.36 mM and −11.15 kcal/mol, respectively.

Among these inhibitors tropolone is the best inhibitor of mushroom tyrosinase with the range of the lowest IC_50_ values of 0.0004–0.0017 mM and binding energy of −4.86 kcal/mol. Ascorbic acid had an IC_50_ value greater than or equal to 0.02 mM, and the IC_50_ value for kojic acid was 0.0074–0.68 mM and 0.04–7.3 mM for arbutin. The binding energy is at −4.63, −4.45, and −4.35 kcal/mol for ascorbic acid, kojic acid, and arbutin, respectively. In the case of human tyrosinase, ascorbic acid had the lowest IC_50_ value and binding energy of ≥0.1 mM and −4.83 kcal/mol. The IC_50_ value and binding energy for kojic acid were 0.50–2.73 mM and −6.00 kcal/mol, values for arbutin were 1.43–6.50 mM and −4.80 kcal/mol, and tropolone had a binding energy of −5.93 kcal/mol. These results correlate with the higher* in vitro* inhibitory activity on human tyrosinase of kojic acid than that of arbutin reported by Kolbe et al. [39].

**Figure 4:**
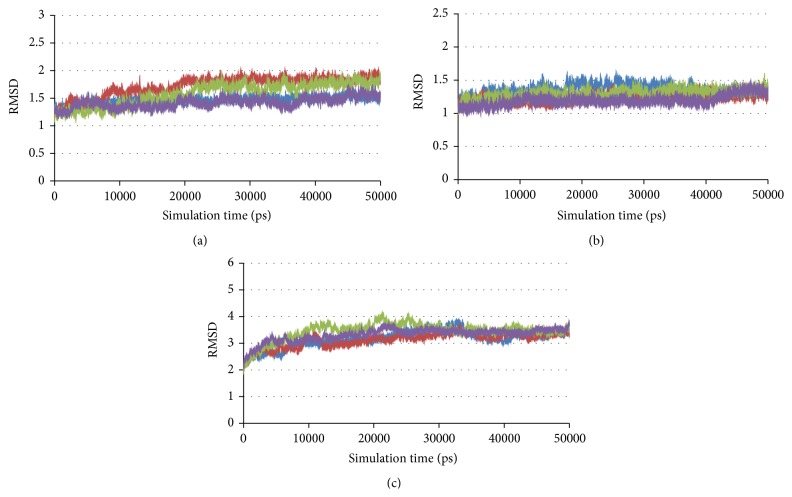
RMSD of carbon backbone in complexes: (a) mushroom tyrosinase-inhibitor complexes, (b) bacterial tyrosinase-inhibitor complexes, and (c) human tyrosinase-inhibitor complexes: arbutin (blue), ascorbic acid (red), kojic acid (green), and tropolone (purple).

To demonstrate attribution of thermal motions on the change of binding site, MD simulations were performed. The binding configuration was rearranged during simulation to observe conformation changes in each time step in comparison with the initial structure. The root mean square deviation of backbone carbon values of the complexes compared with the initial structures is shown in Figure 4.

### 3.3. Intermolecular Interactions and Effectiveness of Specific Inhibition

**Figure 5:**
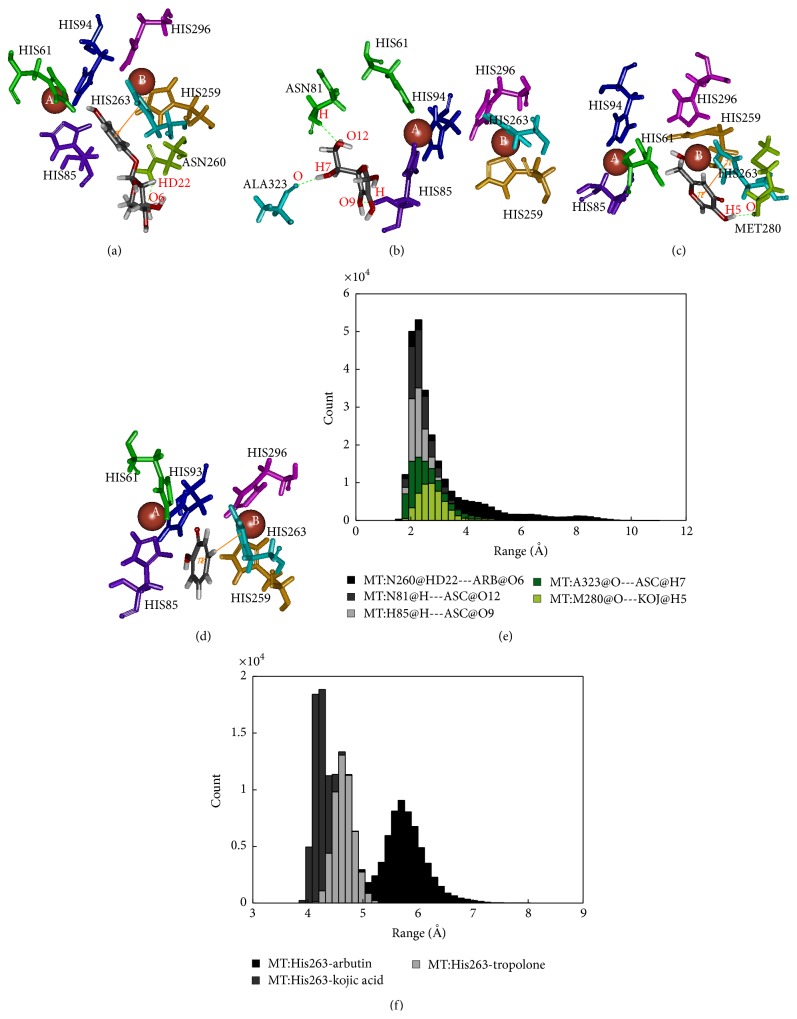
Binding structure of mushroom tyrosinase and inhibitors: (a) arbutin, (b) ascorbic acid, (c) kojic acid, (d) tropolone, (e) distance measurement of hydrogen bond, and (f) distance measurement of pi interaction.

The mushroom tyrosinase-arbutin complex is shown in Figure 5(a). The comparison between a docked and MD structure (Table 2) indicates a decrease in number of hydrogen bonds from 3 bonds with N260, G281, and V283 to only one bond with N260, while pi interaction with H263 is unchanged. Analysis of hydrogen bonding and that of pi distance are shown in Figures 5(e) and 5(f). The results indicate that the mushroom tyrosinase-arbutin complex forms hydrogen bond between N260 and O6:hydroxyl group in part of sugar on arbutin with distance distributed around 2.5 Å and distance distributed between H263 and arbutin interacting using pi-pi interaction around 5.5 Å and had interaction energy of −1.00 kcal/mol. For the mushroom tyrosinase-ascorbic acid complex (Figure 5(b)), three hydrogen bonds on N81 H85, and A323 still remain; thus, the number of hydrogen bonds was decreased from 6 to 3. In this case, the conformation of N81 was altered following the position of arbutin to retain hydrogen bonding.  Figure 5(e) indicated that the mushroom tyrosinase-ascorbic acid complex had 3 hydrogen bonds including amine groups on N81, H85, and carbonyl group on A323 with O12, O9, and H7:hydroxyl group on ascorbic acid, respectively. All of these have distance distributed about 2.5 Å. This result supports the previous study which indicated that ascorbic acid reduces* o*-dopaquinone back to L-dopa, not necessarily at the active site, decreasing melanin formation [46, 47]. The mushroom tyrosinase-kojic acid complex (Figure 5(c)) showed pi interaction with His263 and the hydrogen bonding with M280 was stable. Results from Figures 5(e) and 5(f) indicated that the mushroom tyrosinase-kojic acid complex forms a hydrogen bond between M280 and H5:hydroxyl group on the ring of kojic acid with an intermolecular distance distributed around 3 Å and has pi interactions between H263 and arbutin at 4 Å and had an interaction energy of −0.83 kcal/mol. For mushroom tyrosinase-tropolone complex (Figure 5(d)), the pi interaction with H263 remained stable. The result shows that the mushroom tyrosinase-tropolone complex had pi interaction distance (Figure 5(f)) between H263 and tropolone at 4.5 Å and had interaction energy of −2.68 kcal/mol. According to this result, N81, N260, and M280 are likely to be key amino acids involved in the binding to substrate and H263 forms pi interaction in mushroom tyrosinase. Studies in which N260 and M280 were proposed to play roles in binding substrate [48] and H263 were observed to form pi interaction in mushroom tyrosinase [49].

**Figure 6:**
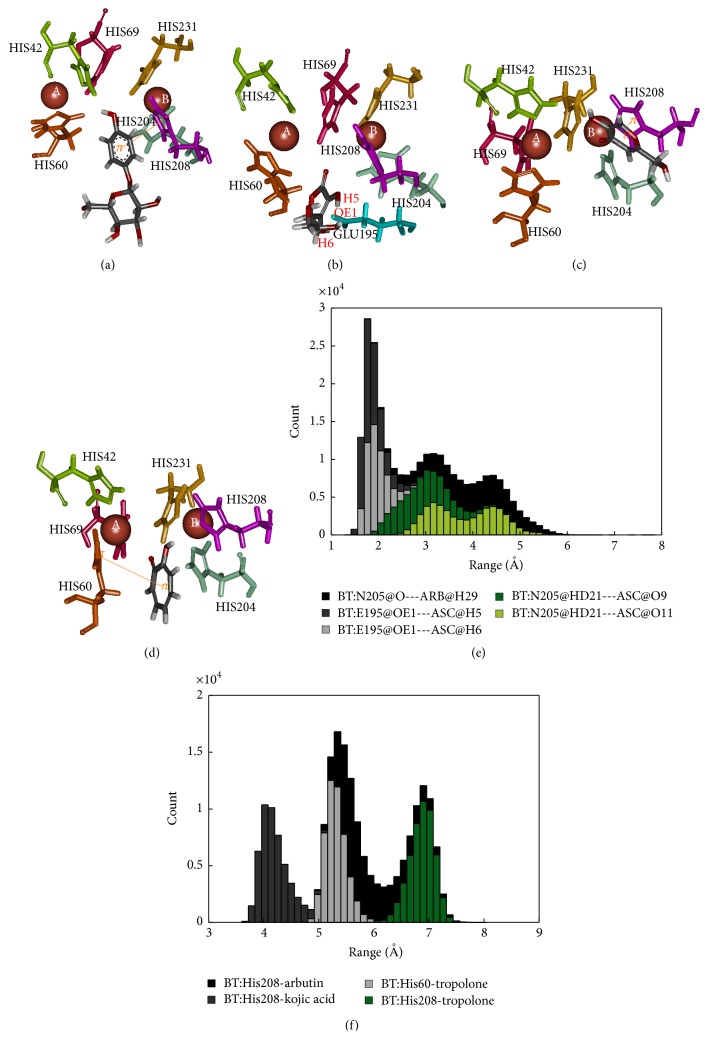
Binding structure of bacterial tyrosinase and inhibitors: (a) arbutin, (b) ascorbic acid, (c) kojic acid, (d) tropolone, (e) distance measurement of hydrogen bond, and (f) distance measurement of pi interaction.

The binding site of the bacterial tyrosinase-arbutin complex is shown in Figure 6(a). The comparison between docked and MD structures (Table 2) indicates that the ligand can retain the pi interaction with H208 while hydrogen bonding is absent. The MD results show the hydrogen bond distance between carbonyl group on N205 and the hydroxyl group in the sugar part of arbutin and revealed the pi interaction distance between H208 and arbutin at 5.5 Å with an interaction energy of −1.84 kcal/mol (Figure 6(f)). For the bacterial tyrosinase-ascorbic acid complex (Figure 6(b)), the number of hydrogen bonds was decreased from 4 hydrogen bonds with H60 and E195 to only 2 hydrogen bonds with E195. The hydrogen bond distance between E195 and H5, H6:hydroxyl group on the ring of ascorbic acid is around 2 Å (Figure 6(e)), indicating a strong interaction. The bacterial tyrosinase-kojic acid complex (Figure 6(c)) had a pi interaction with H208 that is rather stable with distance around 4 Å and had an interaction energy of −4.17 kcal/mol (Figure 6(f)). Hydrogen bonding was not observed in MD, but 1 hydrogen bond in docked structure was found with H60. For the bacterial tyrosinase-tropolone complex (Figure 6(d)), the pi interaction was observed between H208 and H60. The pi interaction distances between H208 and H60 and tropolone were at 7 and 5.5 Å, respectively, as shown in Figure 6(f). H60 had an interaction energy of −0.52 kcal/mol. According to this result, we suggest that E195 is important in binding to substrate, while H208 plays a role in forming pi interactions in bacterial tyrosinase as these interactions remained stable during simulation. The above-mentioned results correlated well with molecular docking results suggested by Kang et al. [13]. In their work, arbutin was found interacting with N205 and H208 using hydrogen bonding and pi interaction, respectively.

**Figure 7:**
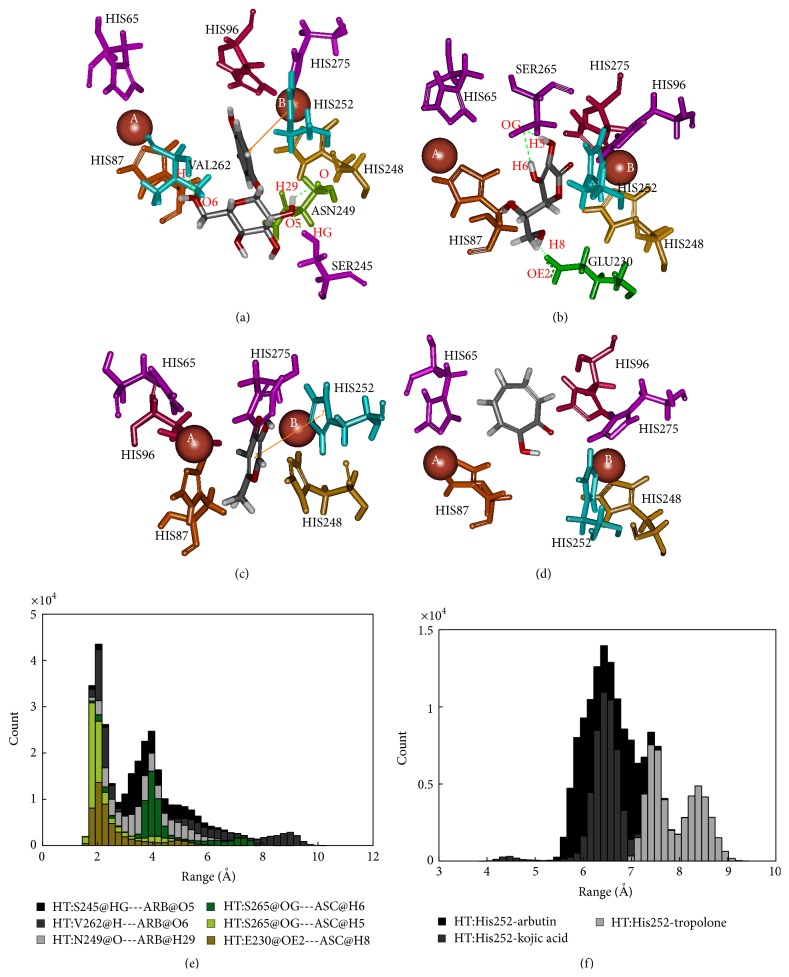
Binding structure of human tyrosinase and inhibitors: (a) arbutin, (b) ascorbic acid, (c) kojic acid, (d) tropolone, (e) distance measurement of hydrogen bond, and (f) distance measurement of pi interaction.

The human tyrosinase-arbutin complex is shown in Figure 7(a). The comparison between the docked and MD structures (Table 2) indicates a decrease from 5 hydrogen bonds with E88, S245, N249, and S265 to 3 hydrogen bonds with S245, N249, and V262 while pi interaction with H252 is unchanged. The hydrogen bonds between S245, V262, and N249 and O5, O6, H29:hydroxyl group in part of the sugar on arbutin were all at approximately 2 Å (Figure 7(e)). The stability of the pi interaction formed in the docked model was analyzed in the MD trajectory in Figure 7(f). The distance between H252 and arbutin was found at 6.5 Å and had an interaction energy of −0.21 kcal/mol. For the human tyrosinase-ascorbic acid complex (Figure 7(b)), a hydrogen bond with E230 still remained. Hence, the number of hydrogen bonds is decreased from 4 to 3. Figure 7(e) shows the hydrogen bond distances between S265 and H18, H20:hydroxyl group on the ring of ascorbic acid, and E230 and H19:hydroxyl group in ring of ascorbic acid, with distances distributed around 2 Å. Binding in the active site of ascorbic acid to tyrosinase was revealed through molecular docking in this study corresponding to previous tyrosinase inhibitory effect proposed by Senol et al. [50]. The human tyrosinase-kojic acid complex (Figure 7(c)) possessed a stable pi interaction with H252 at a distance of 6.5 Å and had an interaction energy of −0.25 kcal/mol (Figure 7(f)). For the human tyrosinase-tropolone complex (Figure 7(d)) neither pi interaction nor hydrogen bonding was found. Figure 7(f) shows a pi interactions distance between His252 and tropolone at 7.5 Å. The results indicated that some residues that are far apart are able to form pi interaction. According to this result, we suggest that E230, S245, N249, V262, and S265 are key amino acids essential in binding to substrate and H252 is involved in forming pi interactions in human tyrosinase.

## 4. Conclusion

In our work, from homology modeling, the 3D structure of human tyrosinase was validated and selected for use in simulation. Binding scaffolds were simulated using molecular docking and molecular dynamics simulation. The binding energy estimated from the simulations was found to be correlated well with the *K*_*m*_ of tyrosinase from the different sources. The obtained structures from docking and MD simulation suggested that N81, N260, and M280 play roles in binding to substrate, and H263 is involved in the formation of pi interactions in mushroom tyrosinase, E195 contributes in binding to inhibitor, and H208 forms pi interaction in bacterial tyrosinase, while E230, S245, N249, V262, and S265 are involved in the binding and H252 is important in forming pi interactions in human tyrosinase.

## Figures and Tables

**Table 1:** Docking score and experimental data in terms of binding structure/activity of tyrosinase from mushroom, bacteria, and human.

Inhibitors/substrate	Mushroom tyrosinase		Bacterial tyrosinase		Human tyrosinase	
	Binding energy	IC_50_/*K*_*m*_	Binding energy	IC_50_/*K*_*m*_	Binding energy	IC_50_/*K*_*m*_
	(kcal/mol)	(mM)	(kcal/mol)	(mM)	(kcal/mol)	(mM)
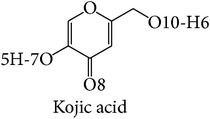	−4.45	0.0074–0.68 [37, 38]	−5.06	—	−6.00	0.50–2.73 [17, 39]

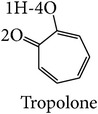	−4.86	0.0004–0.0017 [40, 41]	−4.30	—	−5.93	—

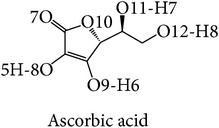	−4.63	≥0.02 [17]	−5.16	—	−4.83	≥0.1 [17]

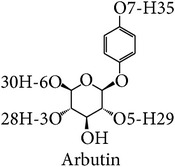	−4.35	0.04–7.3 [42, 43]	−6.09	—	−4.80	1.43–6.50 [17, 37–44]

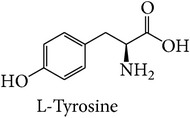	−10.00	0.2 [39] 0.347 [14]	−11.09	0.075 [20]	−11.66	0.17 [2]

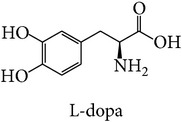	−10.20	0.17 [45] 1.44 [14]	−10.05	0.35 [20]	−11.15	0.36 [2]

**Table 2:** The comparison of interaction site found in docked (in parentheses) and MD structures.

Inhibitors	Mushroom tyrosinase		Bacterial tyrosinase		Human tyrosinase	
	H bonding	Pi interaction	H bonding	Pi interaction	H bonding	Pi interaction
Kojic acid	1:M280 (1:M280)	H263 (H263)	— (1:H60)	H208 (H208)	— (1:N249 2:S265)	H252 (H252)

Tropolone	— (3:N260)	H263 (H263)	— (—)	H60 (H208)	— (2:S265)	— (H252)

Ascorbic acid	1:N81 1:H85 1:A323 (2:N81 4:H85 1:A323)	— (—)	2:E195 (2:H60 2:N205)	— (—)	1:E230 2:S265 (1:Q261 1:V262 2:S265)	— (—)

Arbutin	1:N60 (1:N260 1:G281 1:V283)	H263 (H263)	— (1:N205 1:V218)	H208 (H208)	1:S245 1:N249 1:V262 (2:E88 1:S245 1:N249 1:S265)	H252 (H252)
